# Fabrication and Characterization of Carbon-Fiber-Reinforced Polymer–FeSi Composites with Enhanced Magnetic Properties

**DOI:** 10.3390/polym12102325

**Published:** 2020-10-11

**Authors:** Alexandre Tugirumubano, Sun Ho Go, Hee Jae Shin, Lee Ku Kwac, Hong Gun Kim

**Affiliations:** 1 Institute of Carbon Technology, Jeonju University, Jeollabuk-do 55069, Korea; alexat2020@jj.ac.kr (A.T.); royal2588@naver.com (S.H.G.); kwac29@jj.ac.kr (L.K.K.); 2 Department of Mechanical Engineering, Vision College of Jeonju, Jeollabuk-do 55069, Korea; ostrich@jj.ac.kr; 3 Department of Mechanical and Automotive Engineering, Jeonju University, Jeollabuk-do 55069, Korea

**Keywords:** carbon fiber, magnetic composites, particles, saturation magnetization, permeability, mechanical strength, electrical resistivity

## Abstract

In this work, we aimed to manufacture and characterize carbon-fiber–polymer–metal-particles magnetic composites with a sandwichlike structure. The composites were manufactured by stacking the plain woven carbon fiber prepregs (or carbon-fiber-reinforced polymers (CFRP)) and layers of the FeSi particles. The layer of FeSi particles were formed by evenly distributing the FeSi powder on the surface of carbon fiber prepreg sheet. The composites were found to have better magnetic properties when the magnetic field were applied in in-plane (0°) rather than in through-thickness (90°), and the highest saturation magnetization of 149.71 A.m^2^/kg was achieved. The best inductance and permeability of 12.2 μH and 13.08 were achieved. The composites obviously exhibited mechanical strength that was good but lower than that of CFRP composite. The lowest tensile strength and lowest flexural strength were 306.98 MPa and 855.53 MPa, which correspond to 39.58% and 59.83% of the tensile strength and flexural strength of CFRP (four layers), respectively.

## 1. Introduction

In recent years, carbon-fiber-reinforced polymers (CFRP) composites have continued to emerge in industrial and commercial applications in areas such as aerospace, aircraft, automobiles, transportation, construction, etc. [1,2,3,4,5,6,7] Nowadays, as the interest in those composites continue to grow and they are generally not magnetic materials, it is believed that the application of those composites can be extended if they are magnetically conductive [8,9]. Carbon fibers, owing to their high specific strength, high specific stiffness, good fatigue strength and low density, are commonly used as reinforcing materials in polymer [10] and metal matrix composites [11]. The carbon-fiber-reinforced polymer composites are the main materials used to replace some metallic parts in the various engineering structures while saving the weight of the overall structures.

Different properties of the CFRP composites and their machinability have been extensively studied by various investigators [1,12]. Moreover, the incorporation of other materials to enhance the properties of CFRP composites depending on intended applications have been reported in previous studies. Tomohiro et al. [13] studied the effect of modifying the epoxy matrix using cup-stacked carbon nanotubes (CSCNTs) on the mechanical properties of unidirectional CFRP and quasi-isotropic CFRP composites. The authors demonstrated the improvement of mechanical stiffness, strength, interlaminar fracture toughnesses (mode I and mode II) and a decrease of coefficient of thermal expansion for the unidirectional case. In the case of the quasi-isotropic laminates composite, the CSCNTs were found to improve the compression strength, bending strength, stiffness (in tension, compression, and bending loading) and the compression after impact strengths. They did not find any effect on tensile strength of the composites. Yunsen et al. [14] were able to increase the flexural strength of CFRP composites up to 24.5% at room temperature and up to 30.6% at −100 °C by incorporating the aramid pulp fibers in brittle epoxy matrix. The multiwall carbon nanotubes (MWCNT) were mixed with epoxy resin and the mixture was applied to the carbon fibers fabric in order to reinforce the interface carbon fibers laminar in CFRP composites. That led to the significant improvement in interlaminar fracture toughness of the composites up to 234% and 106% in fracture mode I and mode II, respectively [15]. The reinforcement of iron powder on the interface of carbon prepregs during the CFRP composite forming has resulted in better fracture behavior of the iron–CFRP-particle composites [16].

Halil et al. have found that the dynamic impact damage resistance [17] and the tensile strength [18] of the CFRP composites can be improved by reinforcing then with up to 2 wt.% of alumina (Al_2_O_3_) nanoparticles in an epoxy resin matrix. Ceramic particles (glass flake and aluminum titanate) were coated on the CFRP substrate to act as thermal barrier and to improve the fire performance of the carbon-fiber-reinforced polymer composites [19]. 

Based on the literature [7,12,20,21,22], previous works on CFRP composites have mainly focused on mechanical and structural properties and there is a very limited number of works reporting on the improvement of the magnetic properties of CFRP-based composites. Some of the ways for CFRP composites to become magnetic materials is by either coating the magnetic layer on the carbon fibers [23,24,25,26], introducing the magnetic particles/nanoparticles in a polymer matrix [27,28], or adding the magnetic wires /fibers during the composite manufacturing. 

The electroless coating of magnetic particles such as nickel, nickel-iron, cobalt and ferrite particles on carbon fibers is frequently used as a method to produce magnetic carbon fibers [26,29,30]. The electroless coating method can provide a continuous magnetic layer on the carbon fibers, but this is a time-consuming procedure. Moreover, it involves many chemicals and some considerably expensive chemicals such as PdCl_2_ for coating large parts. The electroplating method [9,31] could be faster and cheaper than electroless coating for generating a thin magnetic layer on carbon fiber, but it can also be time consuming when a thicker layer is needed. Moreover, after both the electroless and electroplating method, the wasted bath solutions have a high risk of environmental pollution [32,33,34].

The mixing of particles with epoxy resin and their application to carbon fiber fabric is another method that is commonly used to produce CFRP-particle hybrid composites [16,20]. The challenge with mixing particles in epoxy resin that usually occurs is to achieve proper dispersion of the particles in the resin as well as in the end composites. Moreover, the ratio of particle-to-epoxy is a limiting factor in the fabrication of the composites [35]. The method that was used in this work involved the distribution of the dry particles on the surface of a carbon-fiber-reinforced polymer (CFRP) prepreg sheet, then covering the layer of particles with another CFRP sheet. In the end product, the particles were bonded to each other by the epoxy resin from the CFRP. This method is easy and fast to produce CFRP-particle hybrid composites and requires very few steps to complete, as is presented in the methods section.

The purpose of this work to manufacture the CFRP-particle hybrid composites with enhanced magnetic properties by stacking the carbon fiber prepregs with layers of magnetic particles. The FeSi powders and carbon-fiber-reinforced polymers (prepreg) (CFRP, plain woven 3K) were used to produce the composites using hot-press compaction. The composites had a sandwichlike composite where the CFRP sheets were used as the skin to provide the mechanical strength and FeSi layers were used as the core to provide the magnetic properties. FeSi was chosen because it is among those iron alloys that have very good magnetic properties such as high saturation induction and high permeability. The morphology, magnetic, mechanical and electric properties of the CFRP-FeSi soft magnetic composites were tested and discussed. It is believed that the procedure used in this work can be used to produce large CFRP-particle composite sheets for practical applications. 

## 2. Materials and Methods

### 2.1. The Preparation and Design of the Composites

The carbon-fiber-reinforced polymers (CFRP prepreg 3K, plain woven, grade: WSN 03KP 200 YS), which are carbon-fiber fabrics preimpregnated with epoxy resin, were supplied by SK Chemicals, Seongnam, Korea. Table 1 shows the typical properties of CFRP prepreg. The FeSi metallic particles (denoted as power flux powders by the manufacture) were purchased from MK Corporation, Incheon, Korea. The specification of the FeSi particles was an average particle size of about 20–30 µm, 4.2% silicon (Si) content and permeability of 90. The FeSi particles were precoated by the manufacture with an electrical insulating layer. 

**Figure 1:**
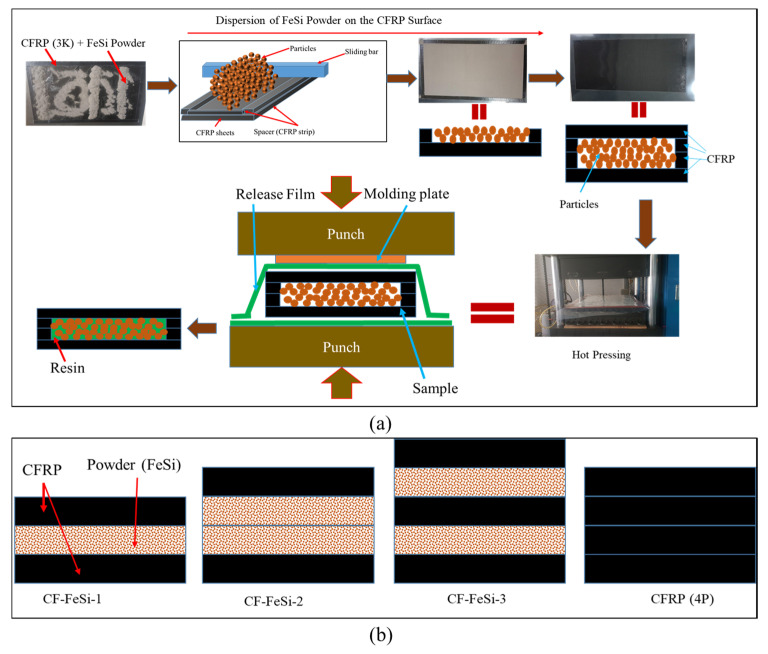
Carbon-fiber-reinforced polymer (CFRP)–FeSi composite fabrication: (**a**) manufacturing procedure; (**b**) CFRP-FeSi lamination designs.

The CFRP sheets were laminated with the layers of FeSi powder, as illustrated in Figure 1. Figure 1a shows the manufacturing process of CFRP-particle composites and Figure 1b presents the lamination sequence of the composite specimens that were considered in this study. In fact, the CFRP prepregs were cut into rectangular sheets. Thereafter, in order to be able to control the thickness of the particle layer during the distribution of particles, small pieces (strips) of CFRP sheets were laminated on the four sides of the larger CFRP sheet prior to particle distribution. Those strips were used to act as spacer layers. As shown in Figure 1a, the dispersion of metallic particles was homogeneously done by sliding the metallic bar on the top of the powder (FeSi) at a height equal to the thickness of the spacer layer. The thickness of the spacer was considered to be equal to one layer or two layers of CFRP sheets. Then, on the top of the distributed particles, another layer of CFRP was added to form a sandwichlike structure. The CFRP layers were oriented in the same direction. The samples of 300 mm length and 160 mm width were considered. The upper surface and lower surface of the stack were covered with the release film so that the resin would not stick to the mold. The upper and bottom punch of the hot press were initially heated to a temperature of 140°C before placing the stacked composite. The stack of CFRP layers and metallic particle layers was then placed in the hot press (Blanket Press, Korea Composite Application (KCA), Gimhae, Korea). The sample was left in contact with the punches of the press for 1 min in order to let the resin flow and fill the gaps between the metallic particles. Then, a pressure of 14 MPa was applied and maintained for 1 h to allow the complete cure of the resin. The produced composite plates were machined into specimens for properties testing. 

### 2.2. The Characterization of the Composites

The morphology of the composites was studied using scanning electron microscope (SEM, CX-200, COXEM Co.Ltd., Daejeon, Korea). The saturation magnetization and coercivity were measured using a vibrating sample magnetometer (VSM 7404-S, Lakeshore Cryotronics, Inc., Westerville, OH, USA). Each VSM sample had size of 5 mm × 5 mm. The applied magnetic field was ±800 kA/m. The magnetic properties measurements using VSM were done by considering the magnetic field travelling in-plane and through-thickness with respect to the specimen. Firstly, the composite sample was attached to the sample holder, which was used for VSM testing using a double-sided stick tape. The in-plane measurement was done by placing and orienting the sample in VSM machine so that the magnetic field of the VSM could flow in a parallel direction with respect to width of the sample (0° direction). For the through-thickness measurement of magnetic properties, the sample was placed in a VSM machine so that the magnetic field flowed across the thickness of the sample (90° direction). This was achieved by setting the head of VSM machine at 0° and 90° for in-plane and through-thickness magnetic properties testing, respectively. 

The inductance, impedance and quality factors were measured using a 300 kHz Bench LCR meter (Model 891, B&K Precision Corporation, Yorba Linda, CA, USA) in the frequency range of 1 kHz to 300 kHz. The later measurements were performed on the single sheet of composite cores. Each core had a 20 mm inner diameter and 33 mm outer diameter. The winding on the core sample was 110 turns of copper magnetic wire (0.4 mm diameter). The permeability of the composites were determined from the inductance data. The electrical resistivity was measured using the four-point probe method with Loresta-GX MCP-T700 (Mitsubishi Chemical Analytech Co., Ltd., Chigasaki, Japan). The mechanical flexural strengths were evaluated on bar specimens (50.8 mm length and 12.7 mm width) in accordance with the standards ASTM D790 [36]. The cross-head speed for flexural testing was calculated for each specimen as specified in the ASTM D790 [36]. Additionally, The mechanical tensile testing was performed on sample with 125 mm length and 12.5 mm width following the standards ASTM D3039 [37]. The cross-head speed for tensile testing was 2 mm/min. The mechanical properties were tested using a universal testing machine, ST-1001 (SALT Co., Ltd., Incheon, Korea).

## 3. Results and Discussion

### 3.1. The Density and Morphology of the Composites

The densities of the composites were calculated from the ratio of mass to volume of the samples. Table 2 presents the density and thickness of each composite sheet. It is evident that the incorporation of particles in CFRP has increased the density. That increase in density can be attributed to the high density of metallic particles.

**Figure 2:**
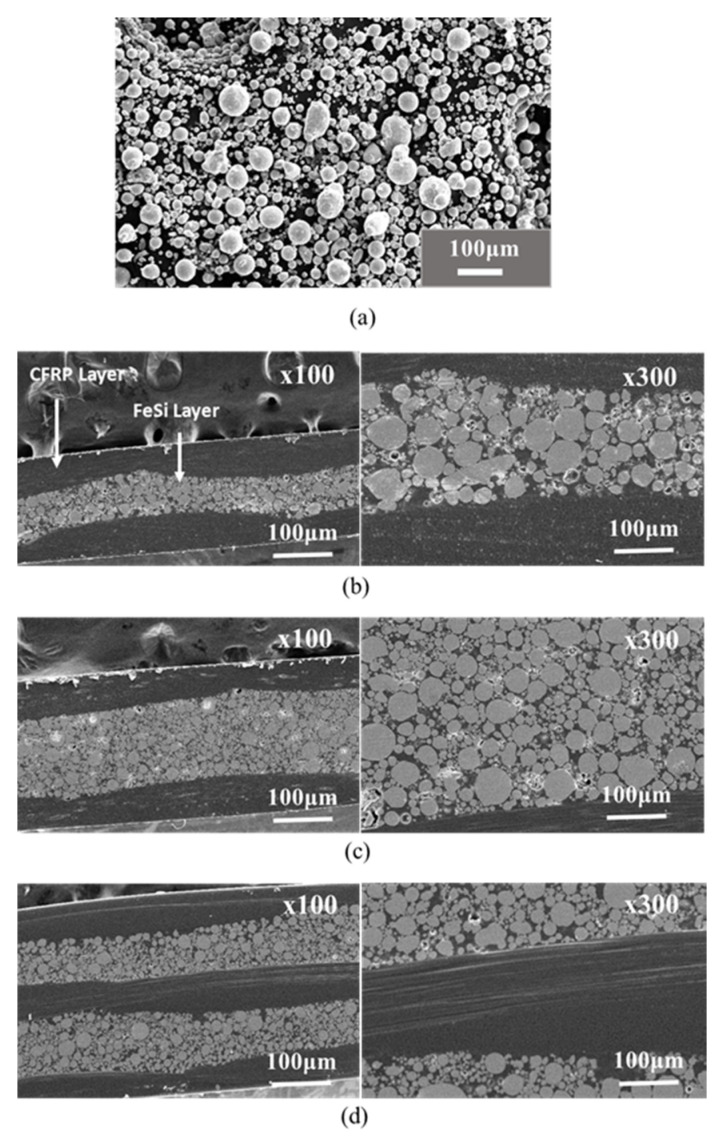
Typical SEM image of CFRP-FeSi composites: (**a**) as-received FeSi powders; (**b**) CF-FeSi-1; (**c**) CF-FeSi-2 and (**d**) CF-FeSi-3.

The morphology of the composites was examined on the polished cross-section of the composites using SEM and the images are shown in Figure 2. It can be seen from SEM images that the epoxy resins have penetrated among the particles to insure the bonding between particles. As a result, the epoxy resins played the role of the matrix for both the carbon fibers and the particles. However, some porosities were observed inside the composites. This can be associated with either the rapid curing of the resin before its complete penetration among the particles or with lower compacting pressure, which was limited by the capacity of the hot press machine. The SEM images, in Figure 2b–d, show that the particles have kept almost their original forms, without significant deformations due to the low compacting pressure.

### 3.2. The Magnetic Properties of the CFRP-FeSi Composites

**Figure 3:**
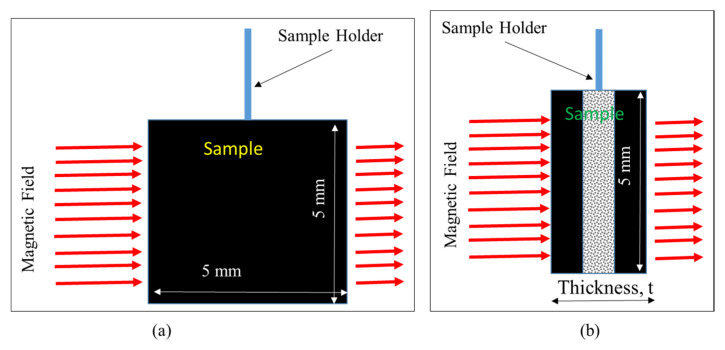
Placement of specimen with respect to magnetic field direction during the VSM measurement: (**a**) field applied parallel to the specimen (in-plane fields); (**b**) field applied perpendicular to the specimen (through-thickness fields).

The magnetic properties of the composites, such as saturation magnetization and coercivity, were evaluated using VSM. The measurements were taken by considering the cases when magnetic field strength was applied either parallel (in 0° direction, in-plane) or perpendicular (in 90° direction, through-thickness) to the specimen as shown in Figure 3. 

**Figure 4:**
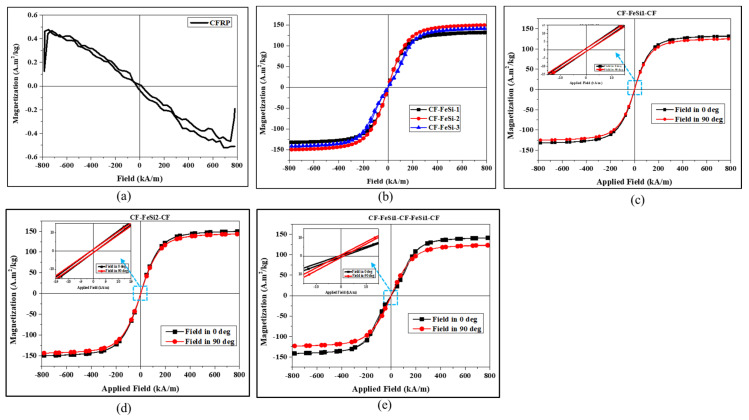
Magnetic hysteresis loops: (**a**) CFRP-pure; (**b**) comparison of hysteresis loops of the CFRP-FeSi soft magnetic composites for the in-plane magnetic field (0°); (**c–e**) comparison of hysteresis loops of CF-FeSi-1, CF-FeSi-2 and CF-FeSi-3 for the field applied in the 0° and 90° directions, respectively.

Figure 4 illustrates the hysteresis loops of the composites. The CFRP composite without magnetic particles exhibited diamagnetic behavior with a negative slope of the hysteresis loop, as shown in Figure 4a. On the contrary, the CFRP-FeSi composites showed the ferromagnetic behavior due to the ferromagnetic particles incorporated in epoxy resin. The saturation magnetization, coercivity and remanence of the materials are given in Table 3. Clearly, the composite CF-FeSi-2 (i.e., CFRP-FeSi2-CFRP) had the best saturation magnetization in all composites. In fact, by increasing the amount of particles in the composite with a limited number of CFRP plies, the magnetic powder-to-epoxy volume ratio was increased and the amount of epoxy to bind the particles was reduced. In addition, the more magnetic particles and less nonmagnetic epoxy were used, the less nonmagnetic space between magnetic particles was formed. This led to the creation of thinner layer around the magnetic particles which could reduce the magnetic reluctance of the composite and promote the transfer of the magnetic field from particle to particle. On the other hand, three plies of CFRP caused a very slight increase in coercivity of the composite. This increase in coercivity can be explained by the increase of impurities and nonmagnetic materials between the ferromagnetic particles as the number of CFRP plies increased. Figure 4b–d and Table 3 show that the saturation magnetization of the composites were generally higher when the composites were placed in parallel with the applied magnetic field (in-plane magnetic field, Figure 3a). For instance, the saturation magnetizations of CFRP-FeSi-2 were 149.63 A.m^2^/kg and 143.55 A.m^2^/kg for the magnetic field strength applied in the 0° direction (in-plane field) and 90° direction (through-thickness field), respectively. That difference in magnetic properties of the composites depending on the direction of the applied field can be attributed to the anisotropic design of the composites. In fact, when the magnetic field was applied in parallel to the sample width, there was a long magnetic path made of continuous FeSi–epoxy layers to carry the magnetic flux. This continuous long magnetic path was advantageous in the enhancement of saturation magnetization. However, when the magnetic field was applied perpendicular to the surface of the sample, there was a very short magnetic path. In addition, the magnetic field was generally interrupted by the nonmagnetic layers (CFRP layers) and, consequently, lower saturation magnetization was recorded. The results in Table 3 show that the orientation of the specimen with respect to applied magnetic field had no significant effect on the coercivity. 

### 3.3. Inductance, Permeability, Impedance and Quality Factor of CFRP-FeSi Composites

**Figure 5:**
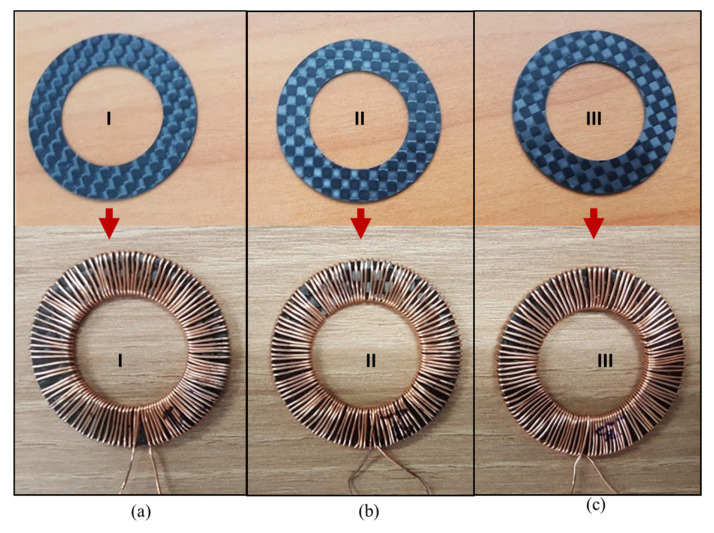
Inductors with CFRP-FeSi core: (**a**) CF-FeSi-1; (**b**) CF-FeSi-3 and (**c**) CF-FeSi-3.

**Figure 6:**
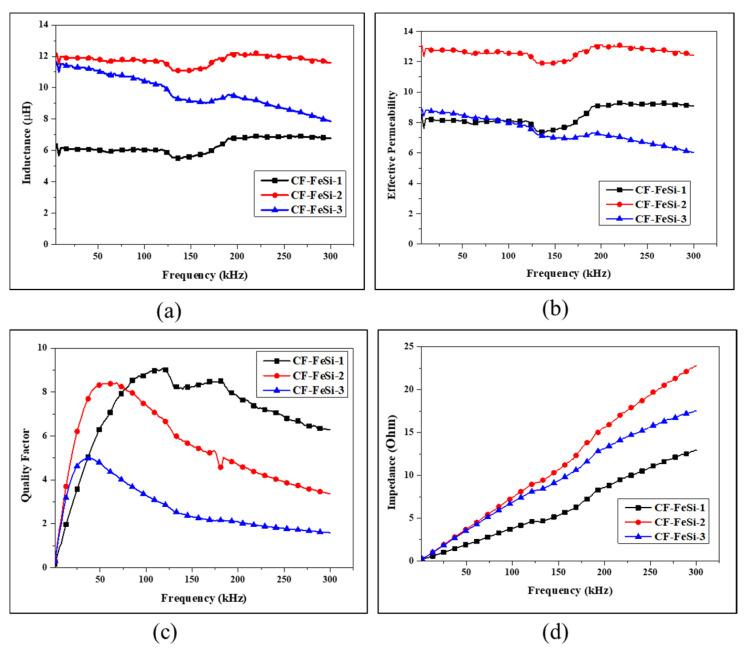
The characteristics of inductors with CFRP-FeSi single-sheet cores: (**a**) inductance; (**b**) effective permeability of CFRP-FeSi cores; (**c**) quality factor and (**d**) impedance.

In order to obtain the permeability of the CFRP-FeSi core, inductors with toroid CFRP-FeSi cores were manufactured. For each CFRP-FeSi magnetic composite, an inductor consisted of a single composite sheet core with a winding of 110 turns of copper magnetic wire of 0.4 mm diameter, as shown in Figure 5. The inductance, impedance and quality factor of the inductors were recorded by considering the series circuit model of the LCR meter [38]. The measurement results, in Figure 6a, indicate that the composite CF-FeSi-2 had the best inductance among the CFPR-FeSi magnetic composites. The maximum inductances were 6.92 µH, 12.2 µH, and 11.6 µH for the composites CF-FeSi-1, CF-FeSi-2 and CF-FeSi-3, respectively. It can be seen that the increase in the volume content of magnetic particles within the composites has contributed to the improvement of the inductance. The effective permeability (µ_eff_) of the each core was calculated from the inductance data by using the following equation [39,40].

(1) $$\mu_{\mathrm{eff}}=\;\frac{{L\;l}_{e}}{4{\;\pi \;N}^{2}A_{e}}$$

where L is the inductance (nH), A_e_ is the effective cross-section area of the core, l_e_ is the effective magnetic path of the core and N is the number of winding turns. The cross-section area [39] and the effective magnetic path [39,41,42,43] were obtained using the following expressions:

(2) $$A_{e}=\frac{(D_{o}-D_{i})t\;}{2}$$

(3) $$l_{e}=\frac{\pi \;\left(D_{o}-D_{i}\right)}{\ln\left(\frac{D_{o}}{D_{i}}\right)}$$

where D_o_ and D_i_ are the outer diameter and inner diameter of the core, respectively, and t is the thickness of the core. 

Figure 6a–b shows that the composite CF-FeSi-2 (i.e., CFRP-FeSi2-CFRP) have both superior inductance and magnetic permeability. This can be attributed to the thicker magnetic layer with relatively lower content of epoxy which formed a thinner insulating layer around the magnetic particles. The greatest permeability of the later composite was 13.05 in the frequency range of 1–300 kHz. The results revealed that the composites CF-FeSi-1 and CF-FeSi-2 have more stability of inductance and permeability than the composite CF-FeSi-3. The instability of the CF-FeSi-3 was indicated by a gradual decrease of its inductance and permeability as the frequency rose. Furthermore, the CF-FeSi-3 had higher permeability than the CF-FeSi-1 at low frequency. However, due to its high instability, its permeability gradually decreased and became lower than that of CF-FeSi-1 at 95 kHz and above. In comparison with other composites, the gradual decrease in permeability of CF-FeSi-3 as the frequency increased can be attributed to the layer of nonmagnetic CFRP stacked between two layers of magnetic particles that led to higher magnetic dilution within the composites [44]. This suggests a considerable increase in the magnetic reluctance of the composite as the frequency rose. 

Figure 6c presents that the quality factors of the composites increased with the frequency to their maxima and then start to gradually drop as the frequency increased.

It is a common knowledge that the quality factor is proportional to the inductive reactance and inversely proportional to the resistance. The reactance is proportional to the inductance and frequency, and inversely proportional to resistance [39,41,42,43,45]. Therefore, it can be inferred that the resistance of the composites may be far greater at higher frequency when compared to the inductive reactance at a given frequency which is higher than the frequency (optimum frequency) of maximum quality factor.

The results, in Figure 6c, show that, in the frequency range of 1–78 kHz, the composite CF-FeSi-2 had the best quality factor. Its maximum quality factor was 8.43 at 68.03 kHz. The composite CF-FeSi-3 had the highest quality factor of 4.98 at 40.03 kHz. For frequencies greater than 78 kHz, the composite CF-FeSi-1 exhibited the best quality factor and its maximum quality factor was 9.07 at 118 kHz. In addition, it was found that, at frequencies greater than 78 kHz, the thicker the composite was (thicknesses are presented in Table 2), the lower was its quality factor. It can be understood that the thicker core will require longer wire windings than the thinner core for a given number of wire turns. The long wire wound on core can produce large distributed capacitances between winding turns, and between winding and core [46,47,48,49]. It also increases the eddy current losses in core windings [50]. In addition, this can promote the increase of skin effects and proximity effects in the windings and core as the frequency increases, and therefore increases the total effective resistance of inductor. Thus, it can be inferred that the combination of the above factors (distributed capacitances, eddy current losses, skin effects and proximity effect), in addition to the hysteresis effect in core composites [51], contributed to the reduction of maximum quality factor with the thickness of the core as the frequency increases. The quality factor expresses the performance of an inductor in terms of converting electric energy and storing it in the form of magnetic energy. The higher quality factor means a better performance of the inductor. Thus, it can be assessed that in frequency range of 78–300 kHz, the thinner core (i.e., CF-FeSi-1) is more effective for storing the electrical energy in the form of magnetic energy. On the other hand, in the frequency range of 1–78 kHz the composite CF-FeSi-2 exhibits better performance.

The continuous increment of the impedance of inductors made with CFRP-FeSi composites when the frequency rose, as shown in Figure 6d. This can be explained by the significant increase in alternative current (AC) resistance of the core and windings as the frequency increases, especially below the self-resonant frequency of the inductor [48,50,52]. The results in Figure 6d indicate that the inductors with a composite core exhibited inductive characteristics in the frequency range of 1 kHz to 300 kHz [52]. 

### 3.4. The Electrical Resistivity of CFRP-FeSi Composites

The measurement of electrical resistivity using the four-probe method showed that the electrical resistivity of the CFRP-particle composites were greater than that the CFRP composite without particle reinforcement, as indicated in Table 4. Their resistivity increased with the thickness of the materials, which is attributed to the large content of FeSi particles and the electrical discontinuity among the adjacent particles due to the epoxy insulating layer around the electrical conductive particles. Therefore, the FeSi particles have improved the electrical resistivity of the composites. In addition, the separation of particles by the nonconductive epoxy resins was essential in enhancing the electrical resistivity of the CFRP-particles composites. The lower resistivity of the CFRP composites may be due to the highly conductive noninsulated carbon fibers that were left on the surface of the composites and the large surfaces of carbon fiber to carbon fiber contacts which were created during the hot pressing of the composites.

### 3.5. The Flexural and Tensile Properties of CFRP-FeSi Composites

**Figure 7:**
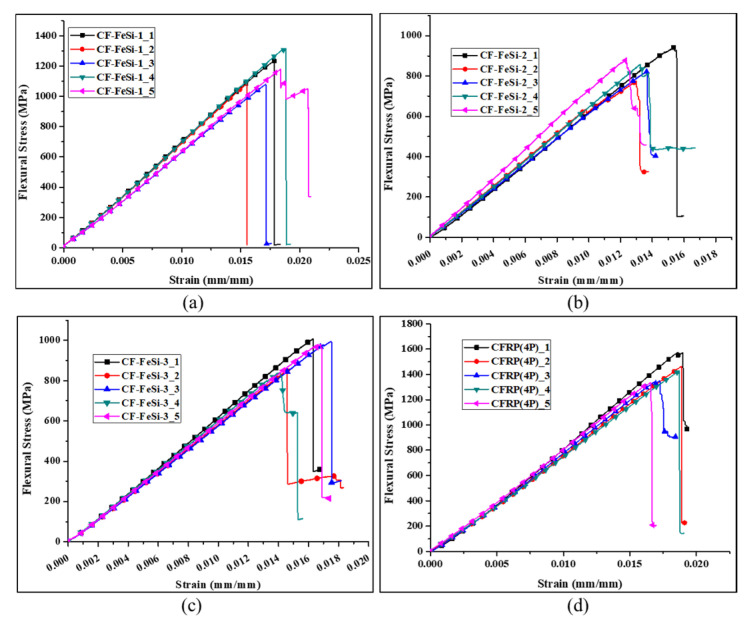
Flexural stress–strain curves CFRP-FeSi soft magnetic composites: (**a**) CF-FeSi-1; (**b**) CF-FeSi-2; (**c**) CF-FeSi-3; (**d**) CFRP (four-ply).

Figure 7 shows the flexural stress–strain curves of the composites. It can be seen that most of the curves dramatically dropped just after reaching the maximum strength. This indicates that the composites are brittle, which can be attributed to the brittleness of the carbon fibers. The flexural strength, tangent modulus of elasticity and chord modulus of all tested specimens for each material and the overall averages are tabulated in Table 5. The chord modulus of each specimen was determined in the strain range of 0.002 mm/mm to 0.006 mm/mm (strain: 0.2 %–0.6%). The particle-free CFRP composite has the best flexural strength of 1429.90 MPa and best chord modulus of 76.90 GPa. The incorporation of FeSi particles in composites greatly reduced the flexural properties (flexural strength, tangent modulus of elasticity and chord modulus). The flexural strength continually decreased when the FeSi content was increased with respect to CFRP content. The results on the flexural toughness which represents the energy absorbed prior to the failure under the flexural test are shown in Table 6. The flexural toughness was obtained by calculating the area under the flexural stress-strain curves of the specimens. It can be seen that the average flexural toughness of each material had a similar trend to the trend of flexural strengths (shown in Table 4). The drop of flexural mechanical and toughness in particle–CFRP composites can be attributed to the poor mechanical properties of particle-epoxy layers, which are brittle. In addition, increasing the amount of particles reduced the volume ratio of high-strength carbon fibers to particles in composites, which caused a drop of flexural strength. As it can be seen in Table 5, CF-FeSi-3 showed better strength than CF-FeSi-2. Although both these composites had the two layers of particle-epoxy, the addition of the CFRP layer in the center of CF-FeSi-3 has increased both the flexural strength and toughness of the composites by 9.24% and 22.92%, respectively. This indicates that the continuous carbon fibers of the CFRP prepeg had a significant impact on strengthening the composites and improving the energy absorption under flexural load.

**Figure 8:**
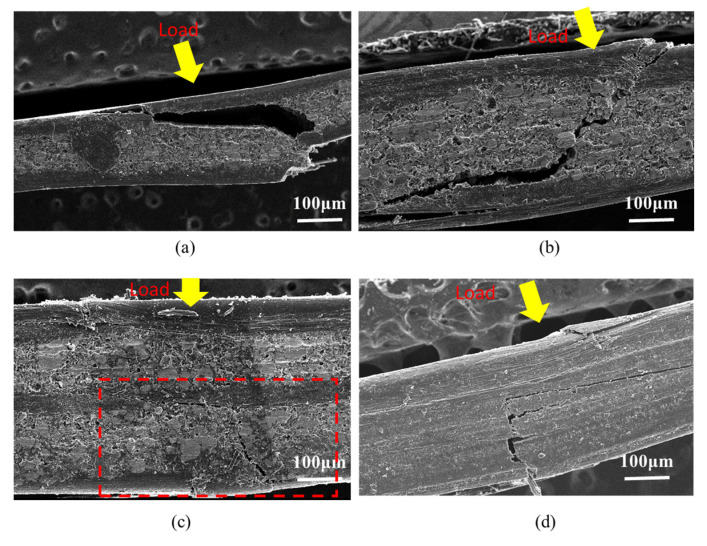
Fractography images of composites after bending test: (**a**) CF-FeSi-1; (**b**) CF-FeSi-2; (**c**) CF-FeSi-3; (**d**) CFRP (four-ply).

Figure 8 shows the fracture behavior of composites after the bending test. The fractography images, in Figure 8, show that the crack propagation in CFRP-FeSi composites was inclined to certain angle with respect to the applied load whereas it has almost the same direction in the case of the CFRP (4P) composite. The crack propagation was obviously not critical in the CF-FeSi-3 sample in comparison with other CFRP-FeSi composites due to the high-strength layer of carbon fibers between the powder layers. This can be attributed to high-strength CFRP layers in the midplane of the CF-FeSi-3 sample.

**Figure 9:**
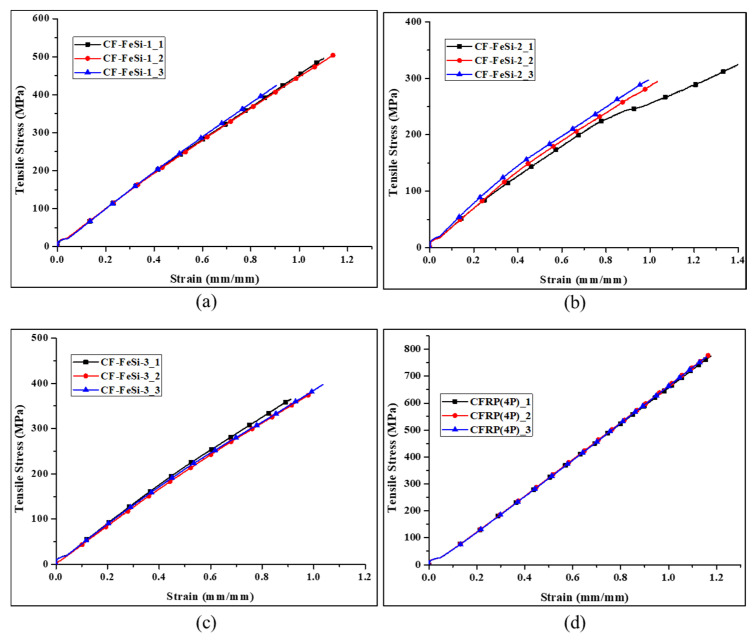
Tensile stress–strain curves of CFRP-FeSi composites: (**a**) CF-FeSi-1; (**b**) CF-FeSi-2; **(c**) CF-FeSi-3; (**d**) CFRP (four-ply).

The tensile stress-strain curves are shown in Figure 9 and the average tensile properties are tabulated in Table 7. The average results are based on three specimens tested for each material. The particulate-CFRP composites had lower tensile strength and lower tensile chord modulus than the four-layer CFRP. The decrease in those mechanical strength due to the incorporation of particles in CFRP might be associated with the insufficient bonding between particles due to low compaction pressure. It can also be attributed to the brittleness behavior and poor strength of FeSi-epoxy layer in the composites. However, the CFRP-FeSi composites had higher Poisson’s ratio than the CFRP composites with four laminated plies. Table 8 presents the tensile toughness of the composites. The calculated averages (Table 7) of tensile toughness for each material show that the composites with high content of particles have lower toughness in comparison to CFRP composite. This can explained by their lower strength and poor ductility. 

**Figure 10:**
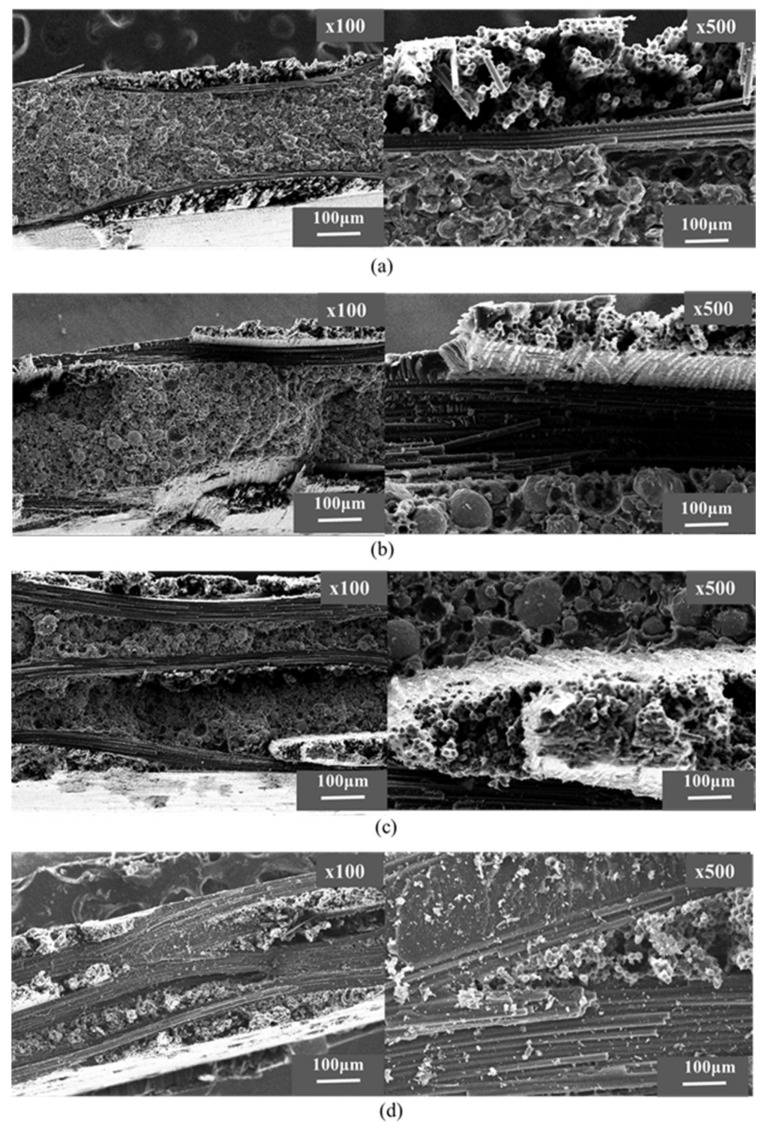
Fractography images of the composites after tensile test: (**a**) CF-FeSi-1; (**b**) CF-FeSi-2; (**c**) CF-FeSi-3; (**d**) CFRP (four-ply).

The fracture behavior of the composites after tensile was examined using SEM and the fractography images are shown in Figure 10. It can be seen that the carbon fibers of the plain woven CFRP oriented in the direction of the applied tensile load and the epoxy-FeSi layers had brittle fracture. The fracture of the epoxy-FeSi particles layers occurred by the detachment of particles from the epoxy matrix and the brittleness fracture of the cured epoxy. Furthermore, there was breakage of some carbon fibers oriented perpendicularly (horizontally) to the applied tensile load. This suggested that the horizontal fibers underwent the bending failure due to the distributed load transferred by the matrix during the testing.

## 4. Conclusions

The CFRP-FeSi magnetic composites were manufactured by stacking the layers of FeSi particles between CFRP layers to form sandwichlike composites. The dispersion of particles of the CFRP plies was performed using a metallic bar to ensure proper distribution of the particles. The composites were compacted using a hot press. 

The morphology study of the cross-section of the composites using SEM showed that the epoxy resins from CFRP had flowed between the particles. That flow of resins guaranteed the bonding between the particles. The composites have shown the ferromagnetic behavior with narrow hysteresis loops, low coercivity and high saturation magnetization of about 149.63 A.m^2^/kg. The good magnetic permeability up to 13.08 and good quality factor 9.07 were achieved. The CFRP-FeSi composites exhibited electrical resistivity greater than 63% compared to CFRP composites without particles, but they had relatively lower flexural strength and tensile strength, lower modulus of elasticity and higher Poison’s ratio when compared with CFRP (four-ply). The improved magnetic properties and electric resistivity of CFRP-FeSi composites suggested that those composites may find potential application in electromagnetic fields for lightweight structures. It is believed that the method used in this study can be used to fabricate particle–CFRP sheets. However, the compacting pressure should be high enough to improve the mechanical strength of the composites. Moreover, the machinability of those composites may be difficult due to the delamination between particles and carbon fibers. 

## Figures and Tables

**Table 1:** Typical properties of carbon-fiber prepreg.

Thickness (mm)	Fiber Areal Weight (g/m^2^)	Weave Density (Warf/Fill)	Resin Content	Fiber Strength (MPa)
0.227	204	12.5/13.5	40%	4900

**Table 2:** Density and thickness of CFRP-FeSi composites.

Composites	Materials ID	Thickness (mm)	Density (g/cm^3^)
CFRP-FeSi1-CFRP	CF-FeSi-1	0.62	3.38
CFRP-FeSi2-CFRP	CF-FeSi-2	0.83	3.76
CFRP-FeSi-CFRP-FeSi-CFRP	CF-FeSi-3	1.06	3.72
CFPR (four-ply)	CFRP (4P)	0.78	1.54

**Table 3:** Magnetic properties of the CFRP-FeSi composites.

Composites	Material ID	Coercivity, Hc (kA/m)		Saturation Magnetization, Ms (A.m^2^/kg)		Remanence, Mr (A.m^2^/kg)	
		Field In 0°	Field in 90°	Field in 0°	Field in 90°	Field in 0°	Field in 90°
CFRP-FeSi1-CFRP	CF-FeSi-1	0.92	0.92	132.22	125.11	0.97	0.92
CFRP-FeSi2-CFRP	CF-FeSi-2	0.92	0.94	149.71	143.55	0.93	0.89
CFRP-FeSi-CFRP-FeSi-CFRP	CF-FeSi-3	1.04	0.94	141.63	123.47	0.47	0.66
CFRP (4P)	CFRP (4P)	-	-	-	-	-	-

**Table 4:** Electrical resistivity of the composites.

Composites	Materials ID	Electrical Resistivity (mΩ.cm)
CFRP-FeSi1-CFRP	CF-FeSi-1	18.88
CFRP-FeSi2-CFRP	CF-FeSi-2	25.84
CFRP-FeSi-CFRP-FeSi-CFRP	CF-FeSi-3	29.74
CFPR (four-ply)	CFRP (4P)	6.90

**Table 5:** Basic flexural properties of the CFRP-FeSi composites.

Materials		Flexural Strength (MPa)		Tangent Modulus of Elasticity (GPa)		Chord Modulus (0.2%–0.6% Strain) (GPa)	
Samples	Specimen No	Each Specimen	Average	Each Specimen	Average	Each Specimen	Average
CF-FeSi-1	1	1233.09	1178.11	67.68	64.96	67.29	64.65
	2	1083.25		67.00		66.70	
	3	1079.21		61.22		60.75	
	4	1316.09		67.70		67.37	
	5	1178.94		61.22		61.14	
CF-FeSi-2	1	944.48	855.53	62.25	64.79	62.28	64.81
	2	773.67		64.40		64.57	
	3	823.47		60.90		60.96	
	4	857.54		63.67		63.70	
	5	878.51		72.72		72.55	
CF-FeSi-3	1	1006.84	934.57	62.70	60.07	62.49	59.95
	2	850.80		58.34		58.38	
	3	994.25		58.32		58.04	
	4	841.16		61.00		60.98	
	5	979.80		59.97		59.85	
CFRP(4P)	1	1574.26	1429.90	81.23	76.91	81.22	76.90
	2	1463.20		74.31		74.37	
	3	1349.85		77.24		77.13	
	4	1425.96		73.17		73.18	
	5	1336.24		78.63		78.60	

**Table 6:** Flexural toughness of the CFRP-FeSi composites.

Specimen No	Flexural Toughness (J/mm^3^)			
	CF-FeSi-1	CF-FeSi-2	CF-FeSi-3	CFRP (4P)
1	11.14	7.41	8.39	15.07
2	8.33	5.51	7.29	13.67
3	9.28	5.87	9.00	12.74
4	12.47	7.37	6.75	13.27
5	13.28	6.34	8.49	11.18
Average	10.90	6.50	7.99	13.19

**Table 7:** Basic tensile properties of the CFRP-FeSi composites.

Materials		Tensile Strength (MPa)		Tensile Chord Modulus of Elasticity (GPa)		Poisson’s Ratio by Chord Method	
Samples	Specimen No	Each Specimen	Average	Each Specimen	Average	Each Specimen	Average
CF-FeSi-1	1	495.73	475.28	48.85	48.90	0.08	0.09
	2	506.06		48.09		0.08	
	3	424.05		49.77		0.11	
CF-FeSi-2	1	329.34	306.98	31.66	33.84	0.07	0.07
	2	294.65		33.65		0.07	
	3	296.95		36.21		0.08	
CF-FeSi-3	1	365.09	380.12	44.02	42.80	0.10	0.10
	2	378.11		41.75		0.09	
	3	397.16		42.64		0.09	
CFRP (4P)	1	774.97	775.59	65.37	65.17	0.05	0.06
	2	780.51		65.44		0.05	
	3	771.28		64.68		0.06	

**Table 8:** Tensile toughness of the composites.

Specimen No	Tensile Toughness (J/mm^3^)			
	CF-FeSi-1	CF-FeSi-2	CF-FeSi-3	CFRP (4P)
1	282.35	271.74	175.27	449.46
2	302.11	165.47	196.25	448.93
3	197.13	162.93	216.96	436.66
Average	260.53	200.04	196.16	445.02
